# Development of a Paper-Based Disposal Thin-Film Solid-Phase Microextraction Tool for the Quantification of Environmentally Hazardous 4-Chlorophenol in Water

**DOI:** 10.1021/acsomega.4c09552

**Published:** 2024-12-20

**Authors:** Harshika Poojary, Partha Pratim Das, Sophia Koo, Chiranjit Ghosh

**Affiliations:** † Department of Biotechnology, Manipal Institute of Technology, Manipal Academy of Higher Education, Manipal, Karnataka 576104, India; ‡ Department of Chemistry, Manipal Institute of Technology, Manipal Academy of Higher Education, Manipal, Karnataka 576104, India; § Division of Infectious Diseases, Brigham and Women’s Hospital, 181 Longwood Avenue, MCP642, Boston, Massachusetts 02115, United States; ∥ Harvard Medical School, 25 Shattuck Street, Boston, Massachusetts 02115, United States; ⊥ Dana-Farber Cancer Institute, 450 Brookline Avenue, Boston, Massachusetts 02215, United States

## Abstract

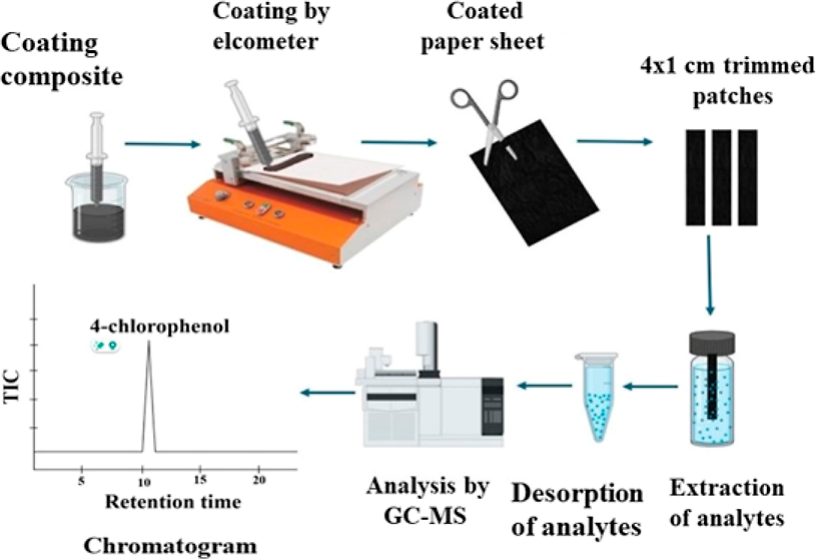

The presence of chlorinated compounds in water resources presents various environmental and health risks. Therefore, there is a precise need to develop a potential technique for fast and efficient monitoring of chlorinated contaminants in water due to environmental protection and regulation compliance. Here, we designed a paper-based thin-film solid-phase microextraction (TF-SPME) patch to estimate 4-chlorophenol (4-CRP), a widely known environmentally hazardous pollutant in water samples. We fabricated the microextraction patch on the paper support utilizing the thin film applicator for uniform coating using divinylbenzene, polydimethylsiloxane, and a multiwalled carbon nanotube (MW-CNT) composite recipe. To check the performance of our fabricated tool, we directly exposed the TF-SPME patches to standard solutions with various concentrations of 4-CRP in water and finally quantified the analyte by exploiting the gas chromatography–mass spectrometer. Our experiments demonstrated the high extraction efficiency of the paper-based TF-SPME analytical tool for the estimation of 4-CRP in water with a limit of detection of ∼10 ng/mL, suggesting the practical applicability of the technique to monitor the analyte within the recommended range. To check the feasibility of the proposed technique for rapid determination, we performed the calibration curve of the analyte in the concentration range of 100–10,000 ng/mL and finally derived the curve fitting equation for the estimation of an unknown amount of 4-CRP. This study demonstrated the feasibility of using a simple paper-based thin-film solid-phase microextraction patch as a sampling kit for monitoring the environmentally hazardous 4-CRP pollutant from water. In the future, the proposed analytical method may be useful for the rapid quantification of chlorinated compounds from the water matrix.

## 1 Introduction

Chlorinated phenolic compounds are common water pollutants originating from various sources, including emissions from petrochemical, pharmaceutical, and textile industrial plants. The use of excessive pesticides in agricultural fields to protect crops from pests enriches the chloroaromatic compounds in water sources.^1,2^ The chlorinated phenolic compounds are also common contaminants produced during pulp bleaching. These contaminants are commonly used as insecticides, fungicides, and disinfectants known for their high toxicity.^3^ The compounds are generally used in the synthesis of agricultural chemicals, biocides, dyes, and pharmaceuticals. Some of the major sources of emission of chlorophenol are oil exploration and production, petrochemical industries, coke oven plants, paper industries (wood pulp treatment through chlorine), pharmaceuticals industries (used as a synthesis intermediate to produce various pharmaceuticals), and paint and solvent industries. They can enter water bodies through industrial effluents, pesticides, and biodegradation of such chlorinated hydrocarbons.^4−6^ The presence of pesticides in water bodies can occur due to various reasons, including agricultural runoff, leaching, and improper disposal. Various chlorophenol derivatives are reported to be poisonous, mutagenic, and even potential carcinogens to living things.^7,8^

Among chloroaromatic compounds, 4-CRP is known as an environmentally toxic compound and a potential candidate for adverse effects on humans, aquatic life, and the environment.^9^ It can cause adverse effects on the human body, like endocrine disruption, reproductive toxicity, and increased cancer risk.^10^ The low water solubility and the highly resistant nature to nonbiodegradation of 4-CRP make it challenging for microorganisms living in the environmental water.^11^ Therefore, it has been classified as a “Priority Pollutant” with a permission limit of 0.5 mg/L in regular potable water.^12^ However, researchers reported the presence of 4-CRP with excess quantities in industry emissions, suggesting the need for the development of a fast and potential method for quantifying 4-CRP from the environmental water^13^

During the past few years, several methods, including solid-phase extraction (SPE) and liquid–liquid extraction (LLE), were developed to detect 4-CRP from water samples.^14−20^ However, the excessive use of organic solvents and, thereafter, the production of a lot of environmentally hazardous byproducts during these conventional sample preparation techniques have limited their applicability as environmentally friendly techniques.^21^

To overcome these limitations, solid-phase microextraction (SPME) was developed by Arthur and J. Pawliszyn in 1990 as a solvent-free and eco-friendly microextraction technique.^22^ The fundamentals of SPME rely on the absorption and desorption techniques of analytes onto a solid-phase coating.^23−25^ The primary advantage of this technique is that it needs a small amount or sometimes no solvent for the analysis of the compounds.^26−29^ Limam et al. used SPME fiber for the quantification of phenol, methyl phenols, chlorophenols, and bisphenol from the water.^30^ However, the practical application of the study was limited due to the fragile nature of SPME fiber. In another study by Ribeiro et al., the SPME was successfully coupled to gas chromatography and mass spectrometry (GC–MS) for the determination of chlorophenol from water through a headspace sampling technique.^31^ The low surface area and fragile geometry of the conventional SPME fiber have limited its widespread application for estimating water pollutants at the trace level. To resolve the issue, researchers developed the thin-film solid-phase microextraction (TF-SPME) technique, another geometry of conventional SPME, with enhanced surface area and flexible geometry for efficient extraction.^32^ TF-SPME utilizes the thermally stable support with uniform coating with polymers for quantitative extraction of the compounds.^33−39^ Although this analytical tool can extract the compounds at parts per billion to parts per trillion levels, the commercial TF-SPME is very expensive and sometimes limits its practical applicability. Therefore, there is a need for the development of an alternative tool for widespread application of the TF-SPME technique for monitoring environmental pollutants.^40−43^

In this research, we proposed a paper-based TF-SPME patch as a flexible analytical sample preparation tool for monitoring 4-CRP from water samples. The objective of this study was to check the feasibility of paper-based TF-SPME for the quantification of 4-CRP through GC–MS analysis. Furthermore, the study was focused on investigating the extraction efficiency of our proposed patches after coating with divinylbenzene (DVB), multiwalled carbon nanotubes (MWCNTs), and polydimethylsiloxane (PDMS) for practical application. However, our proposed paper-based patches may be more cost-effective than commercial TF-SPME patches. Also, the proposed method will need a comparatively lesser amount of solvent consumption than the traditional SPE and LLE methods. Therefore, this paper-based tool may be considered an alternative technique for monitoring environmental pollutants in the future.

## 2 Materials and Methods

### 2.1 Materials

The stock solution of 4-CRP was purchased from Supelco. We used regular commercial A4 size sheet paper (Copy Gold A4 sheet, GSM—75, size 21 × 29.7 cm). Acetonitrile (extra pure, 98%) and hexane were obtained from LOBA Chemicals and Merck, respectively. PDMS, MWCNTs, airtight vials, and 40 mL glass vials with septum and caps were obtained from Sigma-Aldrich. Monomer DVB was purchased from Sigma-Aldrich. The initiator AIBN (98% pure) was acquired from LOBA Chemicals. Cotter pins were purchased from local markets. The commercial DVB/PDMS patches were purchased from MARKES International (Batch number 640689). Parafilm was obtained from Amcor (mode PM-996). All of the glassware was purchased from Borosil. The 40 mL was procured from Sigma-Aldrich.

### 2.2 Instruments

To quantify the pesticide, we exploited a gas chromatograph–mass spectrometer (GC/MS-QP2020 NX SHIMADZU, Shimadzu Corp., Tokyo, Japan). Here, we used an SH-I-5Sil MS column with a 0.25 mm internal diameter and 30 m length. Helium was used as a carrier gas with a consistent column flow rate of 1.2 mL/min. The GC oven temperature was programmed as 50 °C for 2 min, a 6 °C ramp up to 280 °C, followed by a hold for 1 min, a 15 °C ramp up to 320 °C, and finally a hold for 2 min. The ion source temperature (EI) was fixed at 230 °C.

We used an automated film applicator (Elcometer 4340,^44^ Manchester, UK) to perform a uniform coating with a thickness of 70 μm at room temperature. The magnetic stirrer was purchased from REMI (model: 2MLH). The CM-101 Plus vortex shaker was obtained from REMI. REMI CIS-18 Plus, a shaking incubator was utilized during the extraction of compounds from the water matrix. Centrifugation was carried out using an Eppendorf model 5804R centrifuge.

### 2.3 Statistical Analysis

We used OriginPro 2024b (Origin Lab Corporation, USA) software for the analysis of parametric and nonparametric data. In this study, we expressed the data as the mean ± standard error. For the reproducibility of the experimental data at multiple concentration ranges, we plotted calibration curves through the mean data point after three repetitions. A linear fittings plot was constructed to get the fitting equation from the calibration curve, and we evaluated the linear regression data (*R*^2^) from the software.

### 2.4 Synthesis of Coating Materials

#### 2.4.1 Synthesis of the DVB Polymer and Coating Recipe

**Figure 1:**
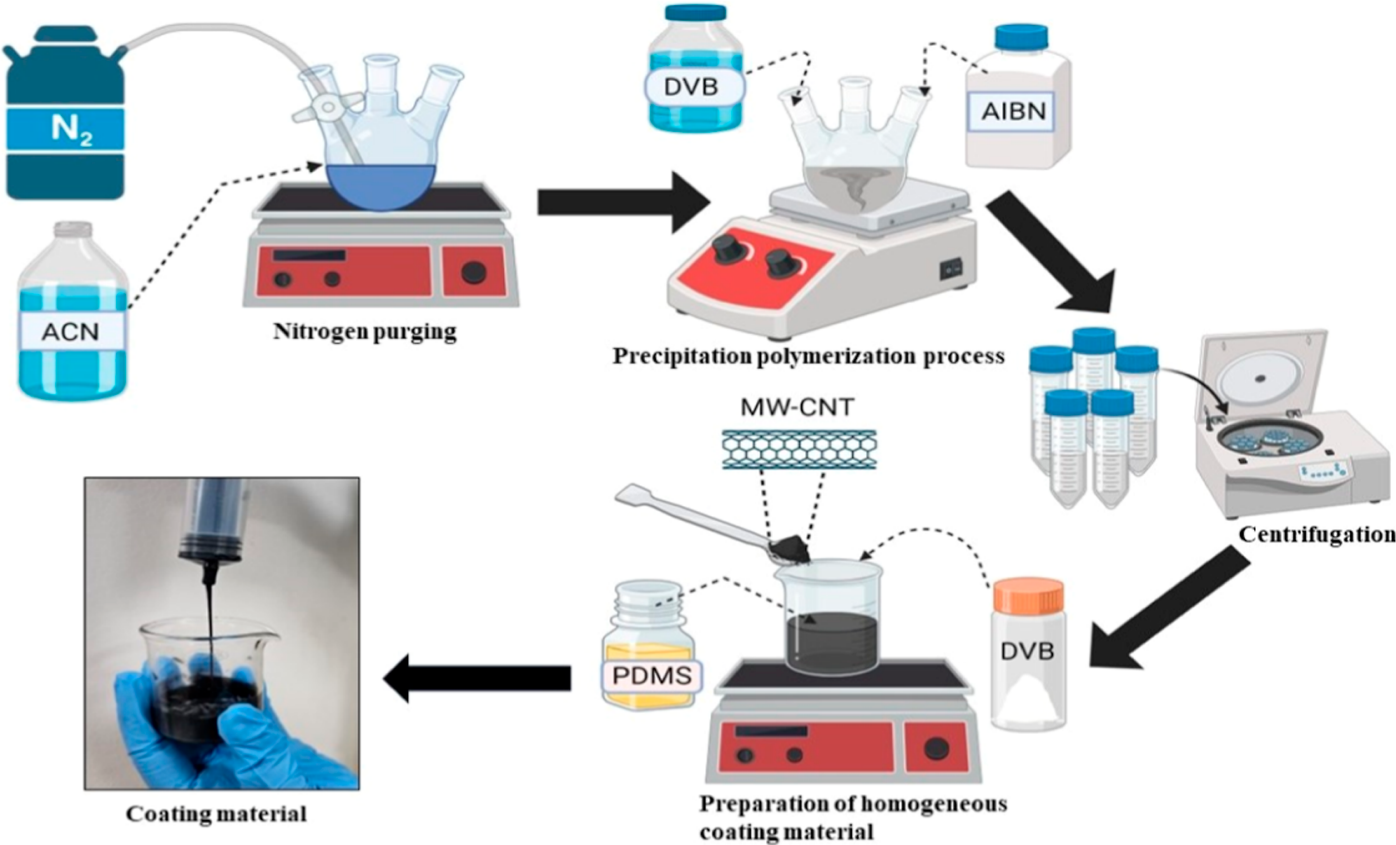
Synthesis of DVB particles by precipitation polymerization and preparation of homogeneous coating recipe with DVB/PDMS/CNT.

DVB polymer was synthesized by the precipitation polymerization process. 400 mL of ACN (acetonitrile) was purged with nitrogen for 2 h in a trinecked round-bottom flask. After purging, 10 mL of monomer DVB and 600 mg 2,2-azobis(isobutyronitrile) (AIBN) initiator were added to the flask. The mixture was stirred at 100 rpm and heated to 75 °C for 24 h. The reaction mixture was centrifuged at 10,000 rpm for 30 min at room temperature. The supernatant was decanted, and the resulting particles were washed with ethanol and vacuum-dried to obtain DVB particles of approximately 1–5 μm in size. Figures 1 and S1a–c depict a brief description of the procedure for the synthesis of coating materials.

To prepare the DVB/PDMS/CNT coating mixture, 1 g of synthesized DVB polymers, 4 mL of PDMS, 0.06 mg of CNT, and 9 mL of hexane were added into the beaker and covered with aluminum foil before placing the composition on a magnetic stirrer for 24 h to obtain a homogeneous mixture.

### 2.5 Fabrication of Paper-Based TF-SPME

**Figure 2:**
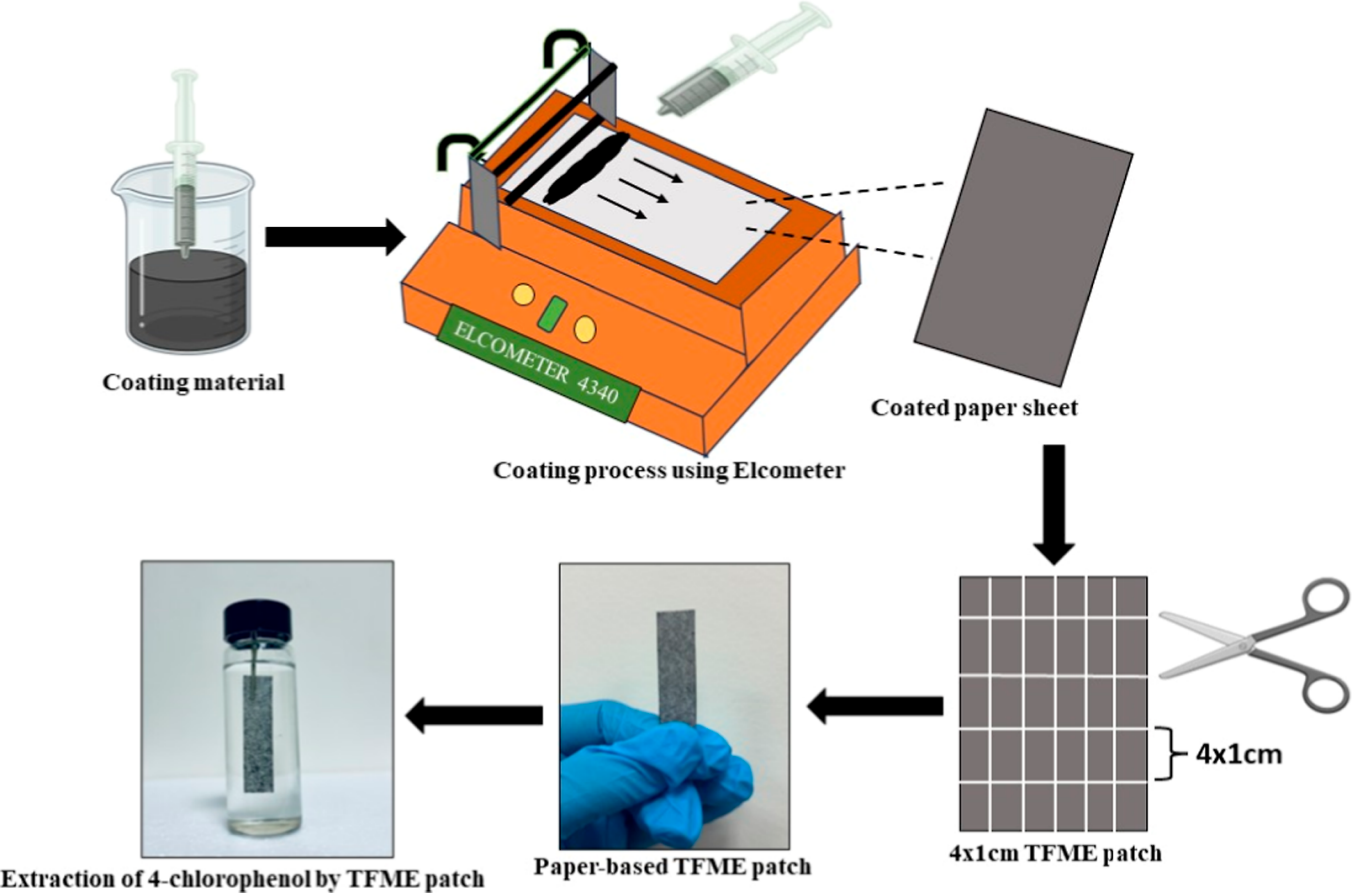
Fabrication of the paper-based TF-SPME analytical tool and extraction of 4-CRP from water.

To prepare the paper-based TF-SPME patches, an A4-size clean paper was cleaned and placed on the automatic film applicator (Elcometer 4340) for coating. To perform the uniform coating, we filled the coating composition into a syringe and finally applied it on to the paper support. The coating thickness was adjusted to 70 μm, and the coating speed was tuned in a way so that we could achieve uniform coating on the paper substrate. After the coating, the whole A4 sheet was air-dried in an oven. We coated both sides of the paper for the efficient extraction of analytes. Finally, the coated A4 sheet was trimmed into multiple 4 × 1 cm patches for use as an individual sampling tool. Figure 2 and Figure S2 show the fabrication of the paper patch after Elcometer coating.

### 2.6 Characterization of the Paper-Based TF-SPME Patches

FE-SEM was carried out by a CARL ZEISS (USA), MODEL: SIGMA WITH GEMINI COLUMN, resolution 1.5 nm using an Inlens detector in field-emission scanning electron microscope. FE-SEM images were acquired in the secondary electron mode at a 10 kV accelerating voltage and at a working distance of 5.5 mm; magnification 10k×; and image resolution, 1 μm. The FE-SEM images were acquired in the secondary electron mode at a 5 kV accelerating voltage and at a working distance of 10.5 mm; magnification 3k×; and image resolution, 10 μm. The elemental composition of the DVB sorbent particles was studied by energy dispersive X-ray spectrometry (EDX)—BRUKER (GERMAN), MODEL: Nano XFlash Detector EDX (elemental analysis): point scan, area scan, line scan, and elemental mapping. Magnification, 2500× *hv*; working distance, 9.8 mm; and image resolution, 20 μm.

### 2.7 Sample Preparation to Extract 4-CRP from Water by Paper-Based TF-SPME

We prepared a main stock solution of 10 ppm of 4-CRP after mixing the compound in water with a magnetic stirrer to ensure no visible particles inside the liquid solution. To perform the extraction of the analyte at various concentrations, we did the serial dilution of the main stock and finally prepared the multiple contraction solution within 100 ng–10,000 ng/mL concentration ranges. We exposed the TF-SPME patch into the sample vials through a cotter pin fixed inside the PTFE material of the cap. These vials were then put into a shaking incubator with a speed of 200 rpm for effective extraction of the pollutants into the paper-based patch. The optimization of the extraction parameters was performed at various stirring rates, temperatures, extraction times, and desorption times. Furthermore, the reusability of our TF-SPME patches was performed with 5000 ng/mL standard solution of 4-CRP. After the extraction, the patch was placed into vials containing 1 mL of acetonitrile (ACN) for desorption. The desorption process was performed using a vortex mixer. GC–MS was used to quantify 4-CRP from the water samples.

## 3 Results and Discussion

**Figure 3:**
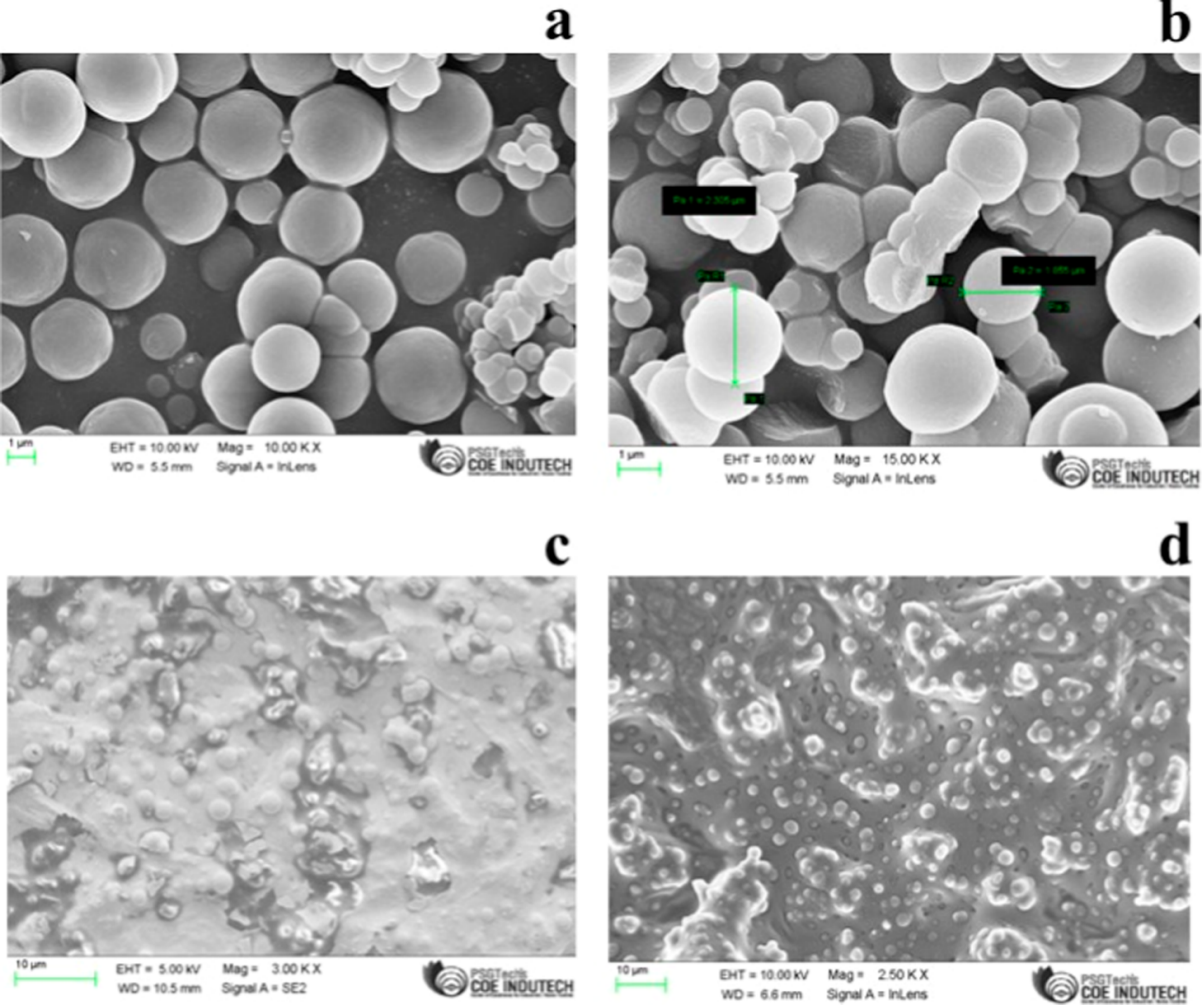
FE-SEM image of the DVB sorbent particles (a and b); (c) FE-SEM image of coated paper-based TF-SPME patch before extraction of 4-CRP; and (d) FE-SEM image of coated paper-based TF-SPME patch after extraction of 4-CRP.

We developed a potential technique exploiting the paper-based TF-SPME analytical tool coupled to GC–MS to quantify 4-CRP from water. To check the particle size of the DVB synthesized in the laboratory, we performed FE-SEM, EDX, XRD, and FT-IR studies. The FE-SEM images of Figure 3a,b demonstrated that the size of our synthesized DVB particles is approximately 1–5 μm in diameter. We performed the SEM and FTIR analysis for the characterization of our fabricated TF-SPME patches.

The FE-SEM images of the coated paper-based TF-SPME patch revealed that our synthesized DVB particles were spherical and had smooth surfaces with uniform distribution, suggesting that the synthesis was well controlled, as shown in Figure 3c,d.

**Figure 4:**
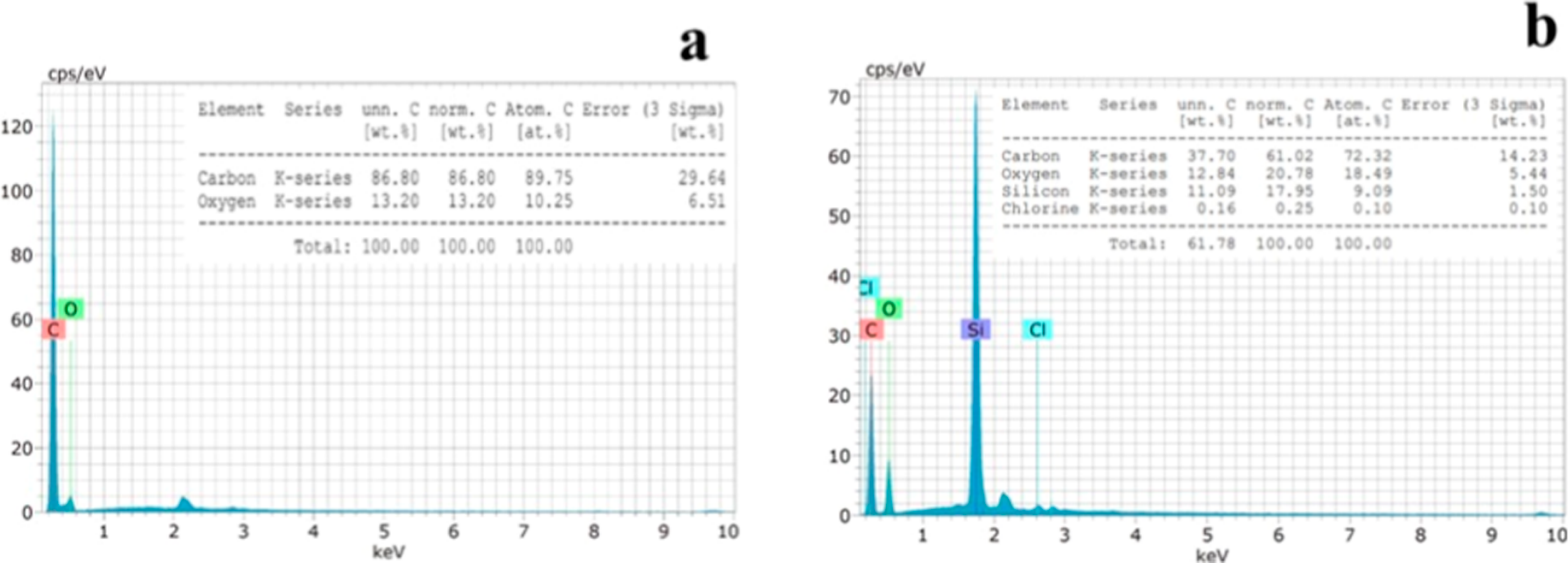
EDX of (a) synthesized DVB particles and (b) DVB/PDMS/CNT-coated TF-SPME patches after extraction of 4-CRP.

The elemental composition of the DVB sorbent particles was studied by EDX, as shown in Figure 4a, indicating that the synthesized DVB particles contained 89.75% carbon and 10.25% oxygen by atomic percentage. In addition, EDX results confirmed that the particles were mostly composed of carbon with trace amounts of oxygen due to surface oxidation or impurities. The EDX data (Figure 4b) of the paper-based patch after extracting 4-CRP showed 72% carbon, 18% oxygen, 9% silicon, and 0.1% chlorine, suggesting adsorption of 4-CRP at a trace level by our developed patch, enabling successful determination of 4-CRP from the water matrix.

**Figure 5:**
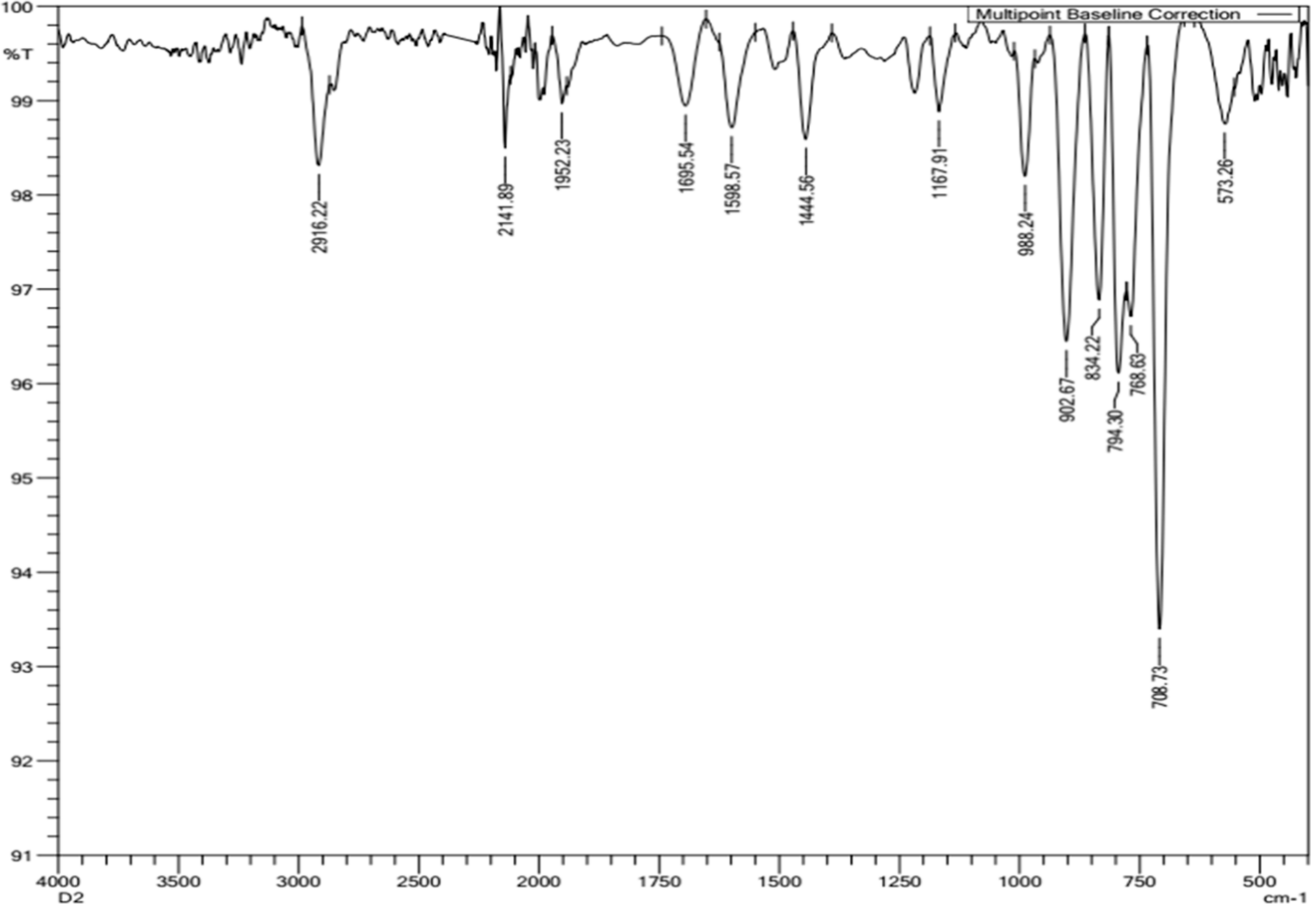
Characterization of DVB particles by Fourier transform infrared spectroscopy.

In the FTIR (Fourier transform infrared) spectrum (Figure 5) of the DVB particles, C–H stretching vibrations (aromatic and vinyl) appear in the region 3100–2900 cm^–1^; C=C stretching vibrations (aromatic) appear in the region 1600–1450 cm^–1^; and C–H bending vibrations (aromatic) appear around 900–700 cm^–1^. These results validate the chemical composition of DVB particles. Minor differences in peak intensities and positions may be attributed to variations in particle size or the presence of impurities.

### 3.1 Stability of Paper-Based TF-SPME

#### 3.1.1 Thermogravimetric Analysis

**Figure 6:**
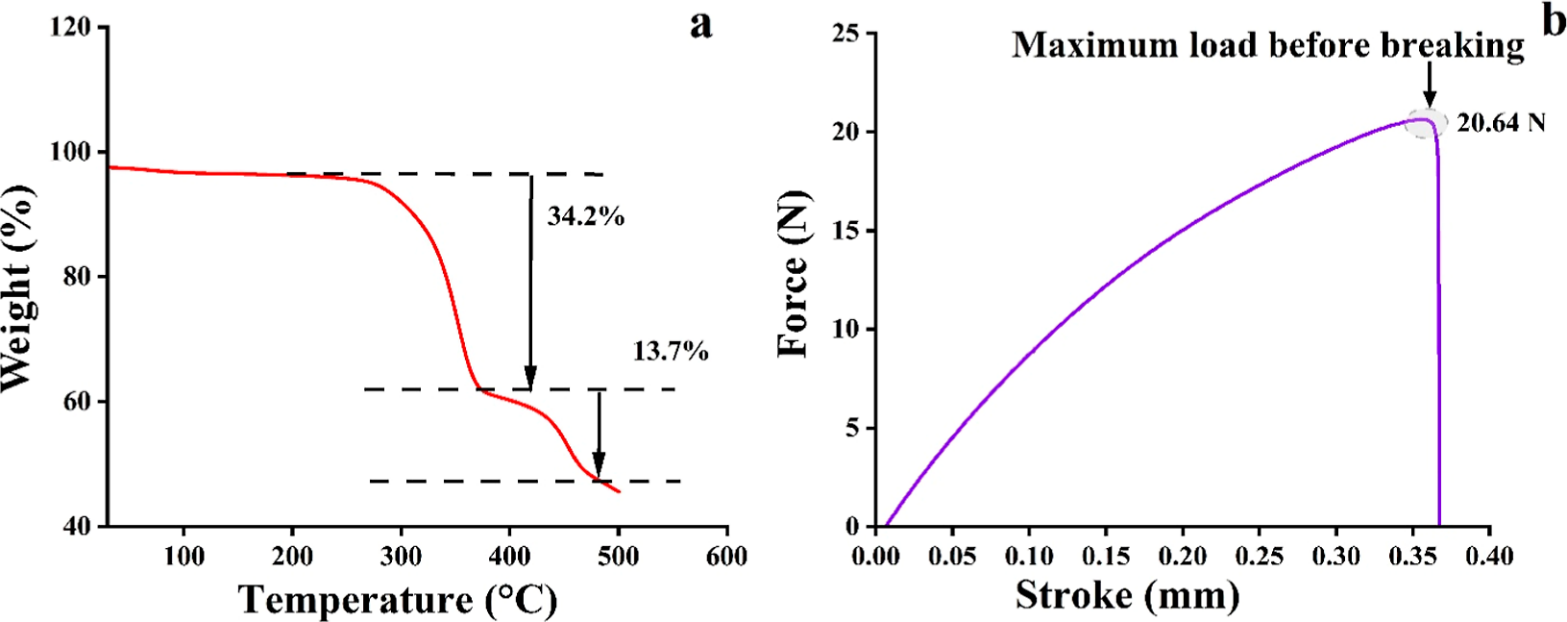
Investigation of the thermal stability of DVB/PDMS/CNT paper-based TF-SPME by TGA (a) and mechanical stability by UTM (b) experiments with DVB/PDMS/CNT-coated paper patches.

To check the thermal stability of the paper DVB/PDMS/CNT patches, thermogravimetric analysis (TGA) was performed with a Hitachi STA7200 equipment within the temperature range of 30–500 °C. The amount of weight loss from the 8 mg patch with the ramp temperature of 5 °C per minute was measured. The thermogram data (Figure 6a) showed that the initial decomposition of the patches started at 230 °C and went up to 350 °C before the second degradation started. However, the primary decomposition of the patches is associated with the moisture content of the patches. Therefore, the TGA analysis suggested that our fabricated paper-based TF-SPME tool was thermally stable up to the temperature of 230 °C, suggesting the potential of our fabricated patches for efficient use in analytical sample preparation purposes.

#### 3.1.2 Universal Testing Machine

To perform the mechanical stability of microextraction patches, the universal testing machine (UTM) test was performed with a Shimadzu-EZ-SX equipment to gain insight into the tensile properties of the materials. We measured the response of our fabricated patch to tension and observed that the maximum tensile strength of the patch was 20.64 N, suggesting considerable stability of the patches for analytical sample preparation applications (Figure 6b).

## 4 Optimization of Various Analytical Parameters for the Extraction of 4-CRP by a Paper-Based TF-SPME Tool

### 4.1 Effect of Extraction Time

**Figure 7:**
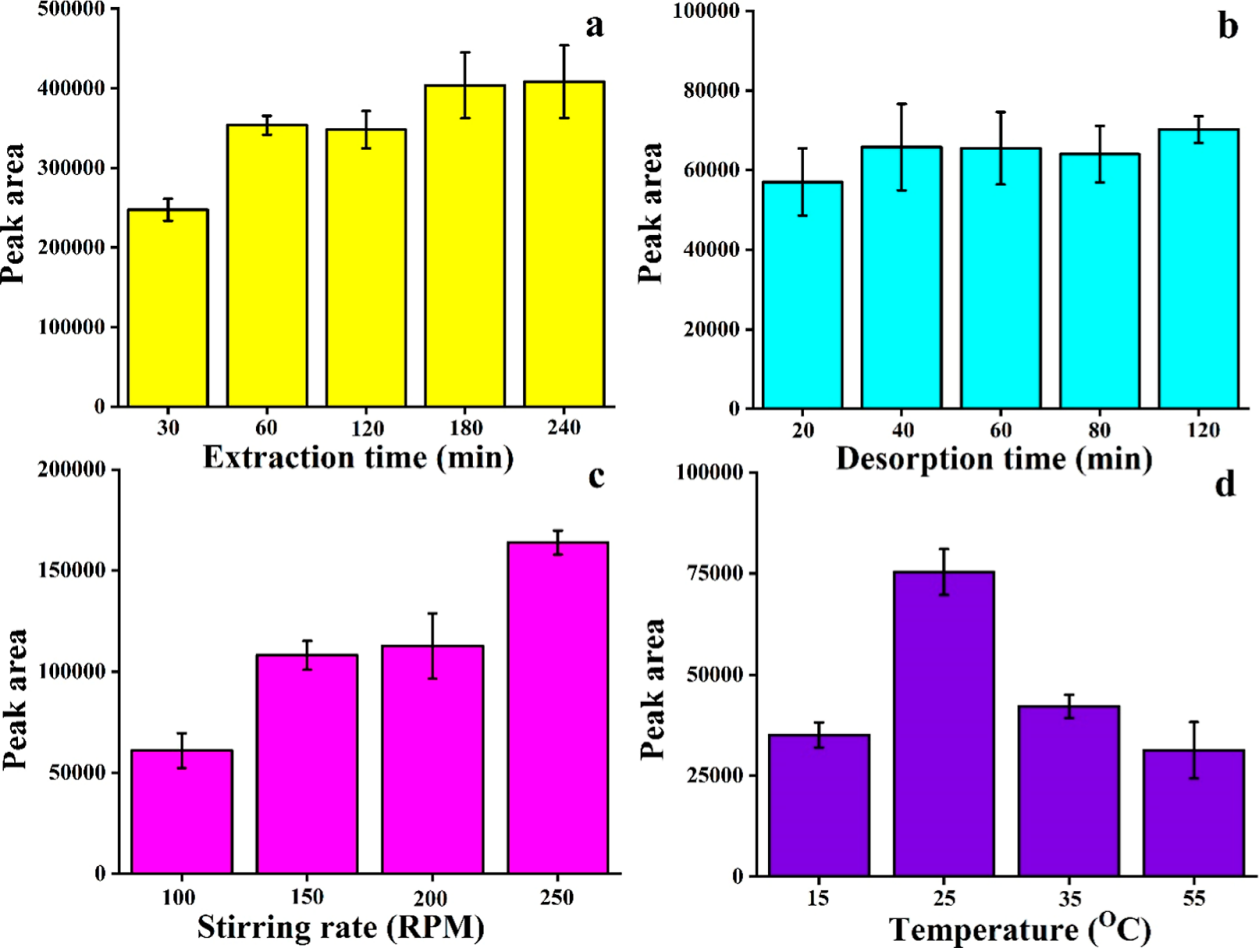
Optimization of various analytical parameters to extract 4-CRP from water samples by paper-based TF-SPME patches, including (a) extraction time, (b) desorption time, (c) stirring rate, and (d) temperature.

The amount of analytes extracted by paper-based TF-SPME is dependent on the extraction time of 4-CRP from the water matrix. In general, the increase in the extraction time facilitates the extraction of more targeted analytes until a plateau is reached at a certain time. Here, we checked the time-dependent extraction efficiency of the patches by individual experiments at various time intervals ranging from 30 to 240 min with 1000 ng/mL 4-CRP in a water sample. Our study showed that the extracted amount of 4-CRP was low at 30 min, and it increased with the enhancement of the extraction time (Figure 7a). However, there is no significant difference in the 180 min and 240 min extraction time profiles, suggesting that the 60 min extraction time is sufficient to get optimal extraction by our paper-based TF-SPME.

### 4.2 Effect of Desorption Time

Desorption time plays an important role in achieving good extraction efficiency, as short desorption time may hamper the complete mass transfer of the targeted analyte from the TF-SPME patch to the solution phase and, finally, may decrease the overall extraction efficiency. The incorporation of the substrate in the coating recipe of TF-SPME with a high surface area and porous structure such as CNT may introduce strong adsorption of analytes on the microextraction patch. In this study, we observed that the 30 min desorption is sufficient to desorb the analytes after extraction from the 1000 ng/mL CRP from water samples (Figure 7b).

### 4.3 Effect of Stirring Rate

The stirring rate plays an important role in extraction efficiency, as the absorption and desorption rates vary with the various stirring rates during the extraction process by the TF-SPME tool. The increase in the stirring rate may facilitate the movement of the 4-CRP compound from bulk to the surface of the TF-SPME tool. Apart from this, the high stirring rate can help to acquire a fast equilibrium and decrease the extraction time. In this study, we performed the extraction with various stirring rates, including 100 rpm, 150 rpm, 200 rpm, and 250 rpm. We observed that an increase in stirring rate significantly increased the extraction efficiency, suggesting the high stirring rate facilitated the diffusion of the 4-CRP effectively into the solvent matrix (Figure 7c). Further enhancement of the stirring rate may damage our paper-based TF-SPME. Therefore, we used the 250 rpm stirring rate for our study to obtain the optimal extraction efficiency.

### 4.4 Effect of Temperature

Thermal energy influences the analyte–sorbent interaction and finally influences the diffusion rate of the analytical technique. The increase in temperature may facilitate the movement of 4-CRP from the sample matrix to the active materials on TF-SPME patches, and this may impact the equilibrium time and absorption process. The desorption process also increases with increases in the temperature. To check the optimal temperature required for the study, we investigated the extraction efficiency of 4-CRP at different temperatures, including 15 °C, 25 °C, 35 °C, 45 °C, and 55 °C. Our result (Figure 7d) demonstrated that we were able to extract the maximum amount of analyte from 1000 ng/mL spiked CRP standard solution at 25 °C temperature.

### 4.5 Reusability Check of Paper-Based TF-SPME Patches

**Figure 8:**
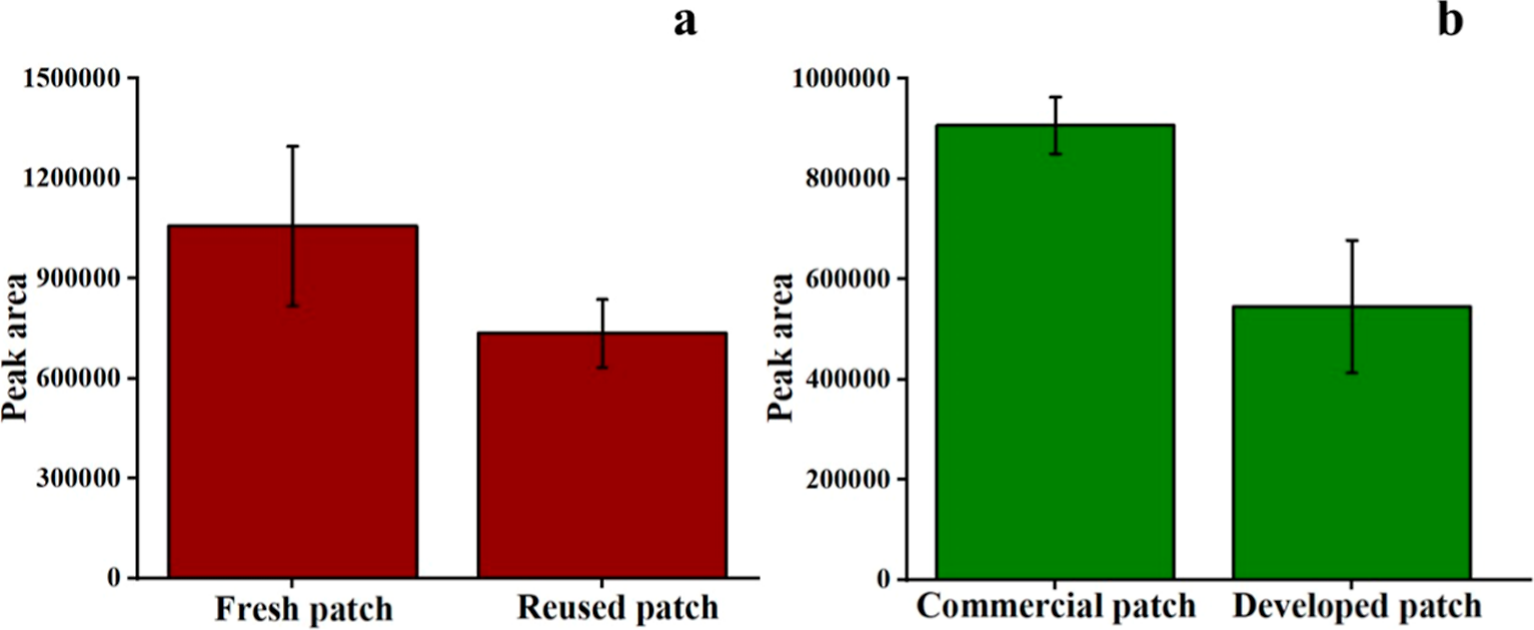
(a) Reusability of paper-based DVB/PDMS/CNT patches; (b) extraction of 4-CRP by paper-based DVB/PDMS/CNT and commercial DVB/PDMS TF-SPME patches.

To investigate the reusability of our proposed TF-SPME patches, we performed the extraction with a 5000 ng/mL standard solution of 4-CRP twice with single patches and five replicates for extraction. After a single experiment, the patches were dried and simultaneously conditioned with nitrogen to remove any residual analytes from the patches. The results (Figure 8a) demonstrated a decrease in extraction efficiency during the second-time application of the patches. Therefore, it may be considered to utilize the paper-based TF-SPME for a single time.

### 4.6 Extraction of 4-CRP by Paper-Based and Commercial TF-SPME Patches

We performed experiments with commercial DVB/PDMS and our proposed DVB/PDMS/CNT-coated TF-SPME patches to check the extraction efficiency of the individual types of patches. We observed the higher extraction efficiency of the commercial patches than our paper-based patches with 5000 ng/mL standard 4-CRP solution in a water matrix (Figure 8b). Although the commercial patches showed high extraction efficiency, those are expensive and are limited for routine environmental applications.

## 5 Extraction of 4-CRP from the Water Matrix

**Figure 9:**
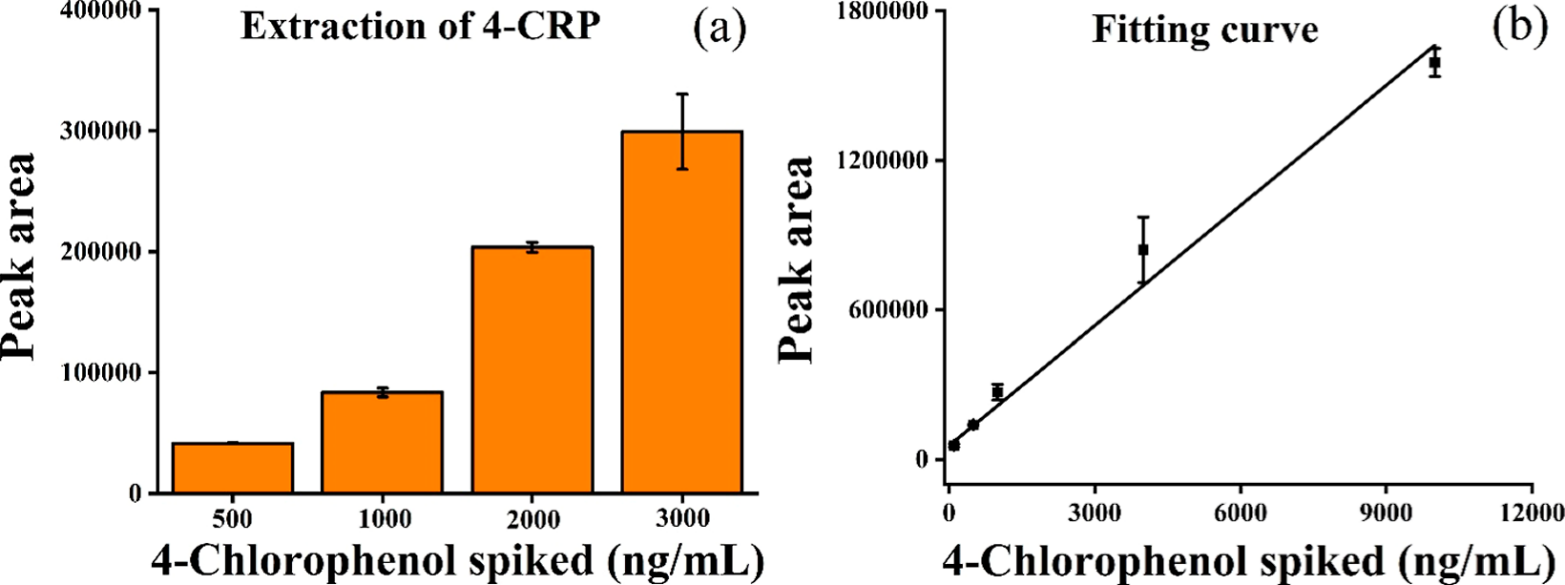
(a) Extraction of 4-CRP from the water matrix; (b) calibration curve of 4-CRP extraction by DVB/PDMS/CNT-coated paper TF-SPME patch. The fitting equation, *y* = 160*x* + 56351, with the linear coefficient value (*R*^2^) is 0.988.

In this study, the paper-based TF-SPME patches were directly immersed into the water samples through a cotter pin inside the vials to extract the 4-CRP compound at various concentration ranges. After extraction, patches were desorbed by a small amount of acetonitrile solvents for mass transfer from the patch to the solution phase, and finally, the samples were analyzed by GC–MS. With a log P value of around 2.39, 4-CRP is moderately hydrophobic, and the hydrophobic nature of CNT coating materials strongly interacts with it, making extraction from the aqueous phase more effective. Our results showed the efficient extraction efficiency of a simple pre-coated paper patch to extract environmentally hazardous pollutants from the water matrix within the concentration range of 100–10,000 ng/mL. Figure 9a represents the bar graph of the peak area vs concentration of 4-CRP. It is evident from the plot that our developed microextraction tool could capture low concentrations of 4-CRP from water.

To check the practical feasibility of this technique, we fitted the calibration curve, and the fitting equation (Figure 9b) was calculated as *y* = *a* + *b* × *x*, where *y* = peak area, *x* = concentration of 4-CRP, *a* = 160, and *b* = 56351. Based on the fitting curve, it is possible to quantify the unknown concentration of 4-CRP from the water matrix using our paper patches. The LOD values were calculated in the range of ∼10 ng/mL. Therefore, it is possible to estimate the unknown amount of 4-CRP from the calibration fitting equation. Once the fitting equation is derived, one can easily quantify the water pollutant without using the conventional SPE sample preparation method for each analysis. Furthermore, to check the efficiency of our proposed method, we spiked the 4-CRP analyte at various concentrations into the water matrix and derived the recovery rate and accuracy parameters. Table 1 shows that the accuracy of the proposed techniques ranged from ∼92% to ∼99%, suggesting the potential efficiency of this technique for practical application.

**Table 1:** Accuracy and Recovery Test after Spiking 4-CRP Standard into the Water Matrix from the Fitting Equation

sample	spiked (ng/mL)	estimation by fitting equation by our proposed method (ng/mL)	accuracy (%)
4-CRP concentration (ng/mL)	500	493	∼98.6%
	1000	1004	∼99.6%
	10,000	9250	∼92.5%

## 6 Conclusions

The present study described the feasibility of paper-based coated patches with nanocomposite coating for the rapid detection of 4-CRP. Our proposed paper-based microextraction patch possesses various advantages, including easy disposable, the requirement of a minimal amount of solvent, and finally, the cost-effectiveness due to the use of regular paper as a substrate for fabricating the tool. Furthermore, the large surface area of the laboratory-based analytical tool was able to detect 4-CRP within the recommended range by various environmental protection agencies. The results showed that our technique was efficient for the accurate determination of the halogenated water pollutants. The limit of detection value was determined within the ∼10 ng/mL range, suggesting feasibility for the measurement of 4-CRP within the recommended threshold value set by the US Environmental Protection Agency and the European Union. The proposed paper-based TF-SPME patches may be more cost-effective than the commercial patches, and this may facilitate practical application for the routine determination of 4-CRP in water as a part of the quality control check. However, our patches are recommended for disposal, whereas the commercial TF-SPME could be used multiple times. Future studies should be performed with analogues of 4-CRP to check any interference of the analogues with the 4-CRP determination. The proposed method may be further validated by assessing and mitigating the interference. The study may help us to understand any deviation in the reported accuracy as mentioned in the manuscript. The proposed approach can be considered a potential and sensitive tool for the rapid quantification of water pollutants. However, more investigation is required to validate the results with the actual water samples contaminated with 4-CRP. Finally, our paper-based patch contributes to sustainable analytical practices and offers a practical solution to the global challenge of contamination of water. In future research, it may be possible to couple the paper-based TF-SPME tool with portable GC–MS for on-site determination of water pollutants.

## Supporting Information Available

Visual representation of the synthesis of divinylbenzene particles by precipitation polymerization and fabrication of paper-based thin-film solid-phase microextraction patches (PDF)

## References

[ref1] Farhod Chasib K. Extraction of Phenolic Pollutants (Phenol and *p* -Chlorophenol) from Industrial Wastewater. J. Chem. Eng. Data 2013, 58 (6), 1549–1564. 10.1021/je4001284.

[ref2] Chi N.; Xu W. Synthesis of TiO2/g-C3N4 Hybrid Photocatalyst and Its Application for Degradation of Chlorophenol as Organic Water Pollutant. Int. J. Electrochem. Sci. 2022, 17 (9), 220929 10.20964/2022.09.10.

[ref3] Zada A.; Khan M.; Khan M. A.; Khan Q.; Habibi-Yangjeh A.; Dang A.; Maqbool M. Review on the Hazardous Applications and Photodegradation Mechanisms of Chlorophenols over Different Photocatalysts. Environ. Res. 2021, 195, 110742 10.1016/j.envres.2021.110742. 33515579

[ref4] Annachhatre A. P.; Gheewala S. H. Biodegradation of Chlorinated Phenolic Compounds. Biotechnol. Adv. 1996, 14 (1), 35–56. 10.1016/0734-9750(96)00002-X. 14536923

[ref5] Henschler D. Toxicity of Chlorinated Organic Compounds: Effects of the Introduction of Chlorine in Organic Molecules. Angew Chem. Int. Ed. Engl. 1994, 33 (19), 1920–1935. 10.1002/anie.199419201.

[ref6] Bhatt P.; Kumar M. S.; Mudliar S.; Chakrabarti T. Biodegradation of Chlorinated Compounds—A Review. Crit Rev. Environ. Sci. Technol. 2007, 37 (2), 165–198. 10.1080/10643380600776130.

[ref7] Olaniran A. O.; Igbinosa E. O. Chlorophenols and Other Related Derivatives of Environmental Concern: Properties, Distribution and Microbial Degradation Processes. Chemosphere 2011, 83 (10), 1297–1306. 10.1016/j.chemosphere.2011.04.009. 21531434

[ref8] Madannejad S.; Rashidi A.; Sadeghhassani S.; Shemirani F.; Ghasemy E. Removal of 4-Chlorophenol from Water Using Different Carbon Nanostructures: A Comparison Study. J. Mol. Liq. 2018, 249, 877–885. 10.1016/j.molliq.2017.11.089.

[ref9] Xin X.; Liu H.; Zhong N.; Zhao M.; Zhong D.; Chang H.; Tang B.; He Y.; Peng C.; He X. A Highly Sensitive Plastic Optic-Fiber with a Molecularly Imprinted Polymer Coating for Selective Detection of 4-Chlorophenol in Water. Sens Actuators B Chem. 2022, 357, 131468 10.1016/j.snb.2022.131468.

[ref10] Velisek J.; Stara A.; Machova J.; Svobodova Z. Effects of Long-Term Exposure to Simazine in Real Concentrations on Common Carp (Cyprinus Carpio L.). Ecotoxicol. Environ. Saf. 2012, 76, 79–86. 10.1016/j.ecoenv.2011.10.013. 22036208

[ref11] Long M.; Zeng C.; Wang Z.; Xia S.; Zhou C. Complete Dechlorination and Mineralization of Para-Chlorophenol (4-CP) in a Hydrogen-Based Membrane Biofilm Reactor (MBfR). J. Clean Prod 2020, 276, 123257 10.1016/j.jclepro.2020.123257.

[ref12] Huang G.-L.; Xiao H.; Chi J.; Shiu W.-Y.; Mackay D. Effects of PH on the Aqueous Solubility of Selected Chlorinated Phenols. J. Chem. Eng. Data 2000, 45 (3), 411–414. 10.1021/je990262k.

[ref13] Igbinosa E. O.; Odjadjare E. E.; Chigor V. N.; Igbinosa I. H.; Emoghene A. O.; Ekhaise F. O.; Igiehon N. O.; Idemudia O. G. Toxicological Profile of Chlorophenols and Their Derivatives in the Environment: The Public Health Perspective. Scientific World Journal 2013, 2013 (1), 460215 10.1155/2013/460215. 23690744 PMC3649668

[ref14] Jiang S.; Li Z.; Yang X.; Li M.; Wang C.; Wang Z.; Wu Q. Sustainable and Green Synthesis of Porous Organic Polymer for Solid-Phase Extraction of Four Chlorophenols in Water and Honey. Food Chem. 2023, 404, 134652 10.1016/j.foodchem.2022.134652. 36283305

[ref15] Tang C.; Tan J. Determination of Chlorophenols in Sewage Sludge and Soil by High-Performance Liquid Chromatography–Tandem Mass Spectrometry with Ultrasonic-Assisted and Solid-Phase Extraction. Anal. Lett. 2017, 50 (18), 2959–2974. 10.1080/00032719.2017.1327537.

[ref16] Ben Hassine S.; Hammami B.; Touil S.; Driss M. R. Determination of Chlorophenols in Water Samples Using Solid-Phase Extraction Enrichment Procedure and Gas Chromatography Analysis. Bull. Environ. Contam. Toxicol. 2015, 95 (5), 654–660. 10.1007/s00128-015-1570-0. 26067701

[ref17] Fattahi N.; Pirsaheb M.; Moradi M.; Mohebbi A.; Karimi P.; Hashemi B. Dispersive Liquid–Liquid Microextraction-Assisted by Deep Eutectic Solvent for the Extraction of Different Chlorophenols from Water Samples Followed by Analysis Using Gas Chromatography-Electron Capture Detection. Microchem. J. 2022, 180, 107608 10.1016/j.microc.2022.107608.

[ref18] Tabaraki R.; Heidarizadi E. Spectrophotometric Determination of Phenol and Chlorophenols by Salting out Assisted Liquid-Liquid Extraction Combined with Dispersive Liquid-Liquid Microextraction. Spectrochim Acta A Mol. Biomol Spectrosc 2019, 215, 405–409. 10.1016/j.saa.2019.02.060. 30870682

[ref19] Chao Y.-Y.; Tu Y.-M.; Jian Z.-X.; Wang H.-W.; Huang Y.-L. Direct Determination of Chlorophenols in Water Samples through Ultrasound-Assisted Hollow Fiber Liquid–Liquid–Liquid Microextraction on-Line Coupled with High-Performance Liquid Chromatography. J. Chromatogr A 2013, 1271 (1), 41–49. 10.1016/j.chroma.2012.11.039. 23237709

[ref20] Sulaiman R.; Adeyemi I.; Abraham S. R.; Hasan S. W.; AlNashef I. M. Liquid-Liquid Extraction of Chlorophenols from Wastewater Using Hydrophobic Ionic Liquids. J. Mol. Liq. 2019, 294, 111680 10.1016/j.molliq.2019.111680.

[ref21] Rodríguez R.; Avivar J.; Leal L. O.; Cerdà V.; Ferrer L. Strategies for Automating Solid-Phase Extraction and Liquid-Liquid Extraction in Radiochemical Analysis. TrAC, Trends Anal. Chem. 2016, 76, 145–152. 10.1016/j.trac.2015.09.009.

[ref22] Arthur C. L.; Pawliszyn J. Solid phase microextraction with thermal desorption using fused silica optical fibers. Anal. Chem. 1990, 62 (19), 2145–2148. 10.1021/ac00218a019.

[ref23] Llompart M.; Celeiro M.; García-Jares C.; Dagnac T. Environmental Applications of Solid-Phase Microextraction. TrAC, Trends Anal. Chem. 2019, 112, 1–12. 10.1016/j.trac.2018.12.020.

[ref24] de Fátima Alpendurada M. Solid-Phase Microextraction: A Promising Technique for Sample Preparation in Environmental Analysis. J. Chromatogr A 2000, 889 (1–2), 3–14. 10.1016/S0021-9673(00)00453-2. 10985530

[ref25] Pawliszyn J. Theory of Solid-Phase Microextraction. In Handbook of Solid Phase Microextraction; Elsevier, 2012; pp 13–59. 10.1016/B978-0-12-416017-0.00002-4.

[ref26] de Fátima Alpendurada M. Solid-Phase Microextraction: A Promising Technique for Sample Preparation in Environmental Analysis. J. Chromatogr A 2000, 889 (1–2), 3–14. 10.1016/S0021-9673(00)00453-2. 10985530

[ref27] Abidi K.; Poojary H.; Keerthana S.; Ghosh C. Solid-Phase Microextraction Techniques for Food Analysis. In Advanced Biophysical Techniques for Polysaccharides Characterization; Elsevier, 2024; pp 235–245. 10.1016/B978-0-443-14042-6.00010-5.

[ref28] Mandal D.; Dey I.; Ghosh C. Development of a Disposable Paper-Based Thin Film Solid-Phase Microextraction Sampling Kit to Quantify Ketone Body. RSC Adv. 2024, 14 (44), 32230–32238. 10.1039/D4RA05907G. 39399252 PMC11469451

[ref29] S K.; Saquib M.; Poojary H.; Illanad G.; Valavan D.; M S.; Nayak R.; Mazumder N.; Ghosh C. Skin Emitted Volatiles Analysis for Noninvasive Diagnosis: The Current Advances in Sample Preparation Techniques for Biomedical Application. RSC Adv. 2024, 14 (17), 12009–12020. 10.1039/D4RA01579G. 38623290 PMC11017966

[ref30] Limam I.; Guenne A.; Driss M. R.; Mazéas L. Simultaneous Determination of Phenol, Methylphenols, Chlorophenols and Bisphenol-A by Headspace Solid-Phase Microextraction-Gas Chromatography-Mass Spectrometry in Water Samples and Industrial Effluents. Int. J. Environ. Anal. Chem. 2010, 90 (3–6), 230–244. 10.1080/03067310903267307.

[ref31] Ribeiro A.; Neves M. H.; Almeida M. F.; Alves A.; Santos L. Direct Determination of Chlorophenols in Landfill Leachates by Solid-Phase Micro-Extraction–Gas Chromatography–Mass Spectrometry. J. Chromatogr A 2002, 975 (2), 267–274. 10.1016/S0021-9673(02)01280-3. 12456081

[ref32] Jiang R.; Pawliszyn J. Thin-Film Microextraction Offers Another Geometry for Solid-Phase Microextraction. TrAC, Trends Anal. Chem. 2012, 39, 245–253. 10.1016/j.trac.2012.07.005.

[ref33] Shahriman M. S.; Mohamad S.; Mohamad Zain N. N.; Raoov M. Poly-(MMA-IL) Filter Paper: A New Class of Paper-Based Analytical Device for Thin-Film Microextraction of Multi-Class Antibiotics in Environmental Water Samples Using LC-MS/MS Analysis. Talanta 2023, 254, 124188 10.1016/j.talanta.2022.124188. 36521327

[ref34] Yiantzi E.; Murtada K.; Terzidis K.; Pawliszyn J.; Psillakis E. Vacuum-Assisted Headspace Thin-Film Microextraction: Theoretical Formulation and Method Optimization for the Extraction of Polycyclic Aromatic Hydrocarbons from Water Samples. Anal. Chim. Acta 2022, 1189, 339217 10.1016/j.aca.2021.339217. 34815047

[ref35] Grandy J. J.; Galpin V.; Singh V.; Pawliszyn J. Development of a Drone-Based Thin-Film Solid-Phase Microextraction Water Sampler to Facilitate On-Site Screening of Environmental Pollutants. Anal. Chem. 2020, 92 (19), 12917–12924. 10.1021/acs.analchem.0c01490. 32847349

[ref36] Murtada K.; Bowman D.; Edwards M.; Pawliszyn J. Thin-Film Microextraction Combined with Comprehensive Two-Dimensional Gas Chromatography Time-of-Flight Mass Spectrometry Screening for Presence of Multiclass Organic Pollutants in Drinking Water Samples. Talanta 2022, 242, 123301 10.1016/j.talanta.2022.123301. 35167962

[ref37] Ripoll L.; Navarro-González J.; Legnaioli S.; Palleschi V.; Hidalgo M. Evaluation of Thin Film Microextraction for Trace Elemental Analysis of Liquid Samples Using LIBS Detection. Talanta 2021, 223, 121736 10.1016/j.talanta.2020.121736. 33298263

[ref38] Olcer Y. A.; Tascon M.; Eroglu A. E.; Boyacı E. Thin Film Microextraction: Towards Faster and More Sensitive Microextraction. TrAC, Trends Anal. Chem. 2019, 113, 93–101. 10.1016/j.trac.2019.01.022.

[ref39] Piri-Moghadam H.; Gionfriddo E.; Rodriguez-Lafuente A.; Grandy J. J.; Lord H. L.; Obal T.; Pawliszyn J. Inter-Laboratory Validation of a Thin Film Microextraction Technique for Determination of Pesticides in Surface Water Samples. Anal. Chim. Acta 2017, 964, 74–84. 10.1016/j.aca.2017.02.014. 28351642

[ref40] Olcer Y. A.; Tascon M.; Eroglu A. E.; Boyacı E. Thin Film Microextraction: Towards Faster and More Sensitive Microextraction. TrAC, Trends Anal. Chem. 2019, 113, 93–101. 10.1016/j.trac.2019.01.022.

[ref41] Murtada K.; Bowman D.; Edwards M.; Pawliszyn J. Thin-Film Microextraction Combined with Comprehensive Two-Dimensional Gas Chromatography Time-of-Flight Mass Spectrometry Screening for Presence of Multiclass Organic Pollutants in Drinking Water Samples. Talanta 2022, 242, 123301 10.1016/j.talanta.2022.123301. 35167962

[ref42] Merlo F.; Profumo A.; Fontàs C.; Anticó E. Preparation of New Polymeric Phases for Thin-Film Liquid Phase Microextraction (TF-LPME) of Selected Organic Pollutants. Microchem. J. 2022, 175, 107120 10.1016/j.microc.2021.107120.

[ref43] Jiang R.; Pawliszyn J. Thin-Film Microextraction Offers Another Geometry for Solid-Phase Microextraction. TrAC, Trends Anal. Chem. 2012, 39, 245–253. 10.1016/j.trac.2012.07.005.

[ref44] Jeżowski P.; Nowicki M.; Grzeszkowiak M.; Czajka R.; Béguin F. Chemical Etching of Stainless Steel 301 for Improving Performance of Electrochemical Capacitors in Aqueous Electrolyte. J. Power Sources 2015, 279, 555–562. 10.1016/j.jpowsour.2015.01.027.

