# Supplementary material for: Development of a Paper-Based Disposal Thin-Film Solid-Phase Microextraction Tool for the Quantification of Environmentally Hazardous 4-Chlorophenol in Water
Source: ACS Omega. 2024 Dec 20;10(1):1657–66. doi: 10.1021/acsomega.4c09552 (PMC11740242; doi:10.1021/acsomega.4c09552)
Supplement: Supplementary file 1 — ao4c09552_si_001.pdf [file ao4c09552_si_001.pdf]

Supporting Information:

Development of paper-based disposal thin film solid-phase microextraction tool for quantification of environmentally hazardous 4-chlorophenol in water

Harshika Poojary[a], Partha Pratim Das[b], Sophia Koo[c,d,e], and Chiranjit Ghosh[a,c,d]*

[a]Department of Biotechnology, Manipal Institute of Technology, Manipal Academy of Higher Education, Manipal, Karnataka, 576104, India.

[b]Department of Chemistry, Manipal Institute of Technology, Manipal Academy of Higher Education, Manipal, Karnataka, 576104, India

[c]Division of Infectious Diseases, Brigham and Women's Hospital, 181 Longwood Avenue, MCP642, Boston, MA 02115, USA

[d]Harvard Medical School, 25 Shattuck Street, Boston, MA 02115, USA

[e]Dana-Farber Cancer Institute, 450 Brookline Avenue, Boston, MA 02215, USA

Corresponding Author: Chiranjit Ghosh **Email: chiranjit.ghosh@manipal.edu**

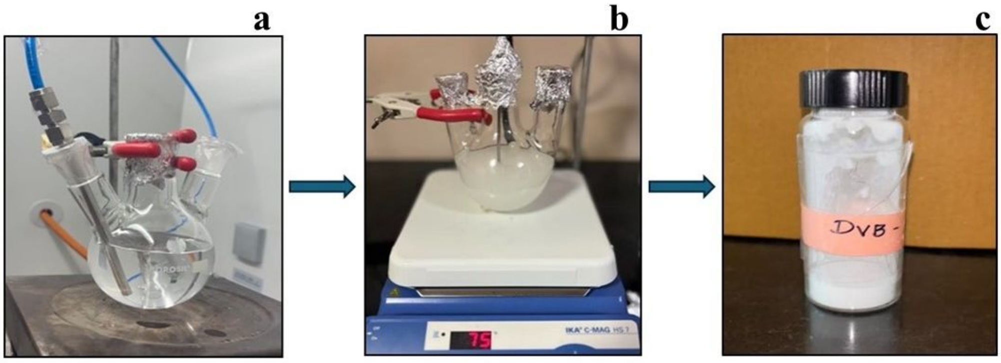

Fig S1 : Synthesis of DVB polymers through steps: (a) purging the round bottom flask containing acetonitrile with nitrogen; (b) precipitation polymerization process after addition of AIBN and monomer DVB; (c) Synthesized DVB polymers

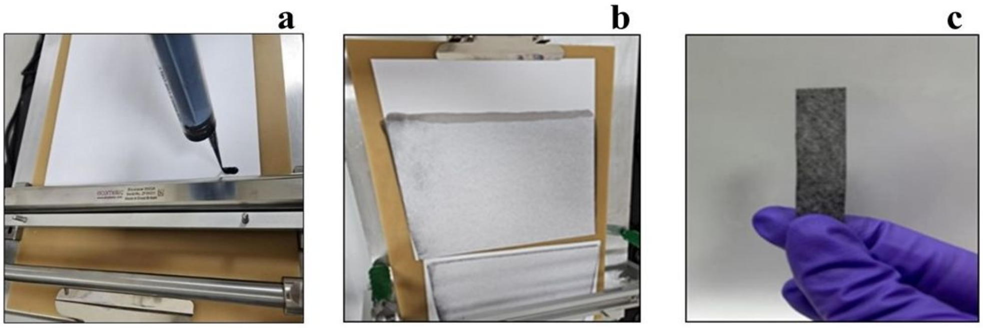

Fig S2: Coating on the paper substrate by Elcometer through steps: (a) application of coating ink onto the paper, (b) post-coated paper TF-SPME, (c) Fabricated TF-SPME patches with 4 cm x 1cm dimension
